# Three-dimensional assembly of multiwalled carbon nanotubes for creating a robust electron-conducting network in silicon-carbon microsphere-based electrodes

**DOI:** 10.1038/s41598-020-58338-3

**Published:** 2020-02-11

**Authors:** Hyemin Kim, Dae-wook Kim, Hitomi Todoki, Nobuyuki Zettsu, Katsuya Teshima

**Affiliations:** 1 Department of Materials Chemistry, Faculty of Engineering, Shinshu University, 4-17-1 Wakasato, Nagano, 380-8553 Japan; 2 Research Initiative for Supra-Materials, Shinshu University, 4-17-1 Wakasato, Nagano, 380-8553 Japan

**Keywords:** Materials science, Materials for energy and catalysis, Batteries

## Abstract

We present a strategic approach to improve the cycle performance of a polymeric binder-free anode based on nano-Si@C microspheres by incorporating a multiwalled carbon nanotubes (MW-CNTs) network and performing carbodiimide-based condensation coupling to form a robust molecular-junction between MW-CNTs and nano-Si@C microspheres. Field-emission scanning electron microscopy reveals that one-dimensional MW-CNTs homogeneously wrapped the individual Si@C microspheres and they interwove through the intergranular nanospace. The incorporation of amide bonds at the junction primarily contributes to the stabilization and reinforcement of the hybrid electrodes. Their reversible capacity after 50 cycles with 0.5 A g^−1^ was significantly improved from 81 mAh·g^−1^ to 520 mAh·g^−1^. Such robustness associated with the supramolecularly assembled MW-CNTs is expected to facilitate electron conductivity and mass transfer kinetics, leading to enhanced electrochemical performance of the Si@C anode.

## Introduction

Silicon is one of the most promising anode materials that delivers a high theoretical capacity of 3574 mAh/g (in association with Li_15_Si_4_ phase formation), which is ten times higher than that of a graphite anode which is widely used in commercialized lithium-ion batteries (LIBs)^1,2^. However, Si anode suffers from massive volume changes during cycling, which results in the pulverization of the active materials of the anode and loss of electrical components. Furthermore, such a huge volume change of the anode causes breakdown and subsequent reformation of the solid electrolyte interface (SEI) layer. These changes lead to a sudden drop in capacity after approximately 10–20 cycles of operation^3–9^.

Various approaches have been proposed to overcome the cyclability issues of a Si anode. For instance, approaches such as the reduction of the Si particle size, adoption of various structural designs, and formation of composites with other components have been widely investigated^10–16^. Composites of Si with carbonaceous materials (e.g., graphene, graphite, and carbon nanotubes etc.) have also attracted much attention, because the carbon materials contribute the physical constrain for volume expansion via covering the silicon crystals. Furthermore, it delivers the decreasing an internal resistance in the electrode via large area direct contact with Si crystal surface^17–19^. Furthermore, many studies have focused on understanding the effect of the structure of polymeric binders on the cycling performances. Nguyen *et al*. systematically studied the effects of binders on silicon anodes by focusing on the SEI formation^20^. Yu *et al*. evaluated the effects of binders on the changes in the electrode thickness during the lithium-ion insertion and extraction processes^4^.

However, previous approaches did not deliver effectively enough for lowering the impedance of the electrodes while simultaneously sustaining their mechanical integrity during the large volume changes for a long cycled operation. The adhesion between the Si particles and polymeric binders is not still sufficiently strong for the fixation of Si particles via physisorption, leading to the detachment of the coating and capacity fading upon cycling.

Very recently, we proposed a new strategic approach based on a supramolecularly assembled cathode composed of multiwalled carbon nanotubes (MW-CNTs) and LiNi_0.5_Co_0.2_Mn_0.3_O_2_ (NCM523) for achieving both high rate capability and stable cycling characteristics at a high tapped density of 3.8 g cm^−3^ ^21,22^. Through supramolecular assembly of the electrode components, a stable conductive network can be formed inside the electrode and the insulating polymer binder can be excluded from the interior parts of the electrode. The MW-CNT stretched reversibly with changes in the volume of the NCM523 particles, and rendered the electrode highly robust to resist damage with long-term cycling.

Herein, we expanded the aforementioned strategy to an anode based on nano-Si crystals embedded in a carbon microsphere (Si@C) using supramolecular MW-CNTs as the binder. Particularly, since the Si anode undergoes a larger volume change than a NCM523 cathode during cycling, the effect of molecular bonding between the MW-CNT binder and Si@C microsphere was examined. We comparatively analyzed three different hybrid electrodes with different interactions: (i) CNT/Si@C with van der Waals interaction, (ii) CNT/NH_2_-Si@C with attractive electrostatic interaction, and (iii) CNT/1-(3-Dimethylaminopropyl)-3-ethylcarbodiimide hydrochloride (EDC)/NH_2_-Si@C with covalent bonding (amide bond). Furthermore, we did not use a polymeric binder to more clearly verify the effect of chemical bonding in our electrode system.

## Results and Discussion

### Preparation of CNT/Si@C hybrid systems

**Scheme 1:**
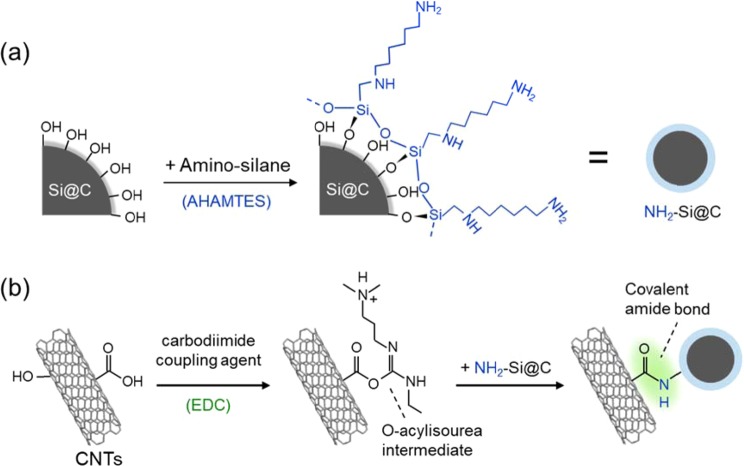
Schematic illustration of the procedure for the preparation of the covalently cross-linked CNT/EDC/NH_2_-Si@C electrode; (**a**) surface silanization of Si@C and (**b**) carboxyl group (–COOH) activation for spontaneous reaction with a primary amine (–NH_2_) on Si@C using EDC as a coupling agent.

**Figure 1:**
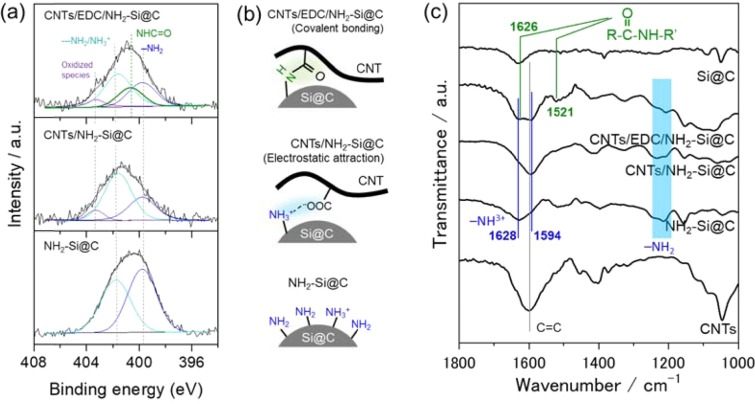
(**a**) XPS N 1 s core-level spectra and (**b**) illustrations of NH_2_-Si@C (bottom), CNT/NH_2_-Si@C (middle), and CNT/EDC/NH_2_-Si@C (upper) systems. (**c**) FT-IR spectra of CNT/Si@C hybrid composite systems used in the preparation of electrodes.

The hybrid of MW-CNTs and Si@C microspheres was prepared in three steps by solution processes, as shown in Scheme 1. Chemical states and local structures of the series of Si@C microsphere-based electrodes, including CNT/EDC/NH_2_-Si@C, CNT/NH_2_-Si@C, and NH_2_-Si@C were studied by XPS and ATR-FT-IR spectroscopies to track the coating process. Figure 1a shows the XPS N 1 s core-level spectra of the three materials. Spectral features of primary amine (–NH_2_, 399.7 eV) and hydrogen bonded/protonated amine (–NH_2_/NH^3+^, 401.7 eV) groups suggest that primary amine groups in the AHAMTES monolayer were densely immobilized on the Si@C microsphere surface through the silane coupling reaction. Since it is well known that silane coupling agents readily react with hydroxyl groups on metal oxide surfaces, the results indicate the presence of a native oxide layer on the nano-Si crystal surface within the top-layer of soft-carbon layer that is accessible for the silane coupling reactions. The peak intensity of the primary amine decreased with no appearance of new peaks in the XPS of the mixture of NH_2_-Si@C and MW-CNTs (CNT/NH_2_-Si@C). This result indicates that, at the interface, MW-CNT network and Si@C microsphere interacted through electrostatic interactions without any chemical bonding. In contrast, a new intense peak centered at 400.6 eV attributed to amide bonds (–NH–C=O) appeared in the XPS of CNT/EDC/NH_2_-Si@C, implying that EDC cross-linking agent promoted the amide bond formation between the MW-CNTs and NH_2_-Si@C microsphere surface (Fig. 1b).

Further, we conducted FT-IR spectroscopy to identify the condensation reaction between the MW-CNT and Si@C microsphere assisted by EDC coupling gent. In Fig. 1c, new signals appeared at 1650–1500, 1240–1040, 990–880, and 770–750 cm^−1^ in Si@C microsphere after surface modification with AHAMTES monolayer. Furthermore, signals in the range of 1650–1500, 1240–1040, and 770–750 cm^−1^, which can be attributed to protonated amine (–NH^3+^), primary aliphatic amine (–NH_2_), and secondary aliphatic amine (–NH–) groups, were also detected. These results strongly support the functionalization of the Si@C microsphere surface with AHAMTES molecules. Note that the area of the peaks of free amine (–NH_2_) at 1240–1020 cm^−1^ decreased significantly, while that of the peaks at ~1650 and 1035 cm^−1^ attributed to C=C and C=O in the MW-CNTs increased, suggesting the binding of NH_2_-Si@C microspheres to MW-CNTs through electrostatic attractive interaction. Moreover, CNT/EDC/NH_2_-Si@C complex prepared using the EDC coupling agent showed two distinct IR peaks at 1626 and 1521 cm^−1^, assigned to amide bonds (R–CO–NH–R′). These FT-IR spectral features are consistent with the XPS spectral characteristics of the materials shown in Fig. 1a.

**Figure 2:**
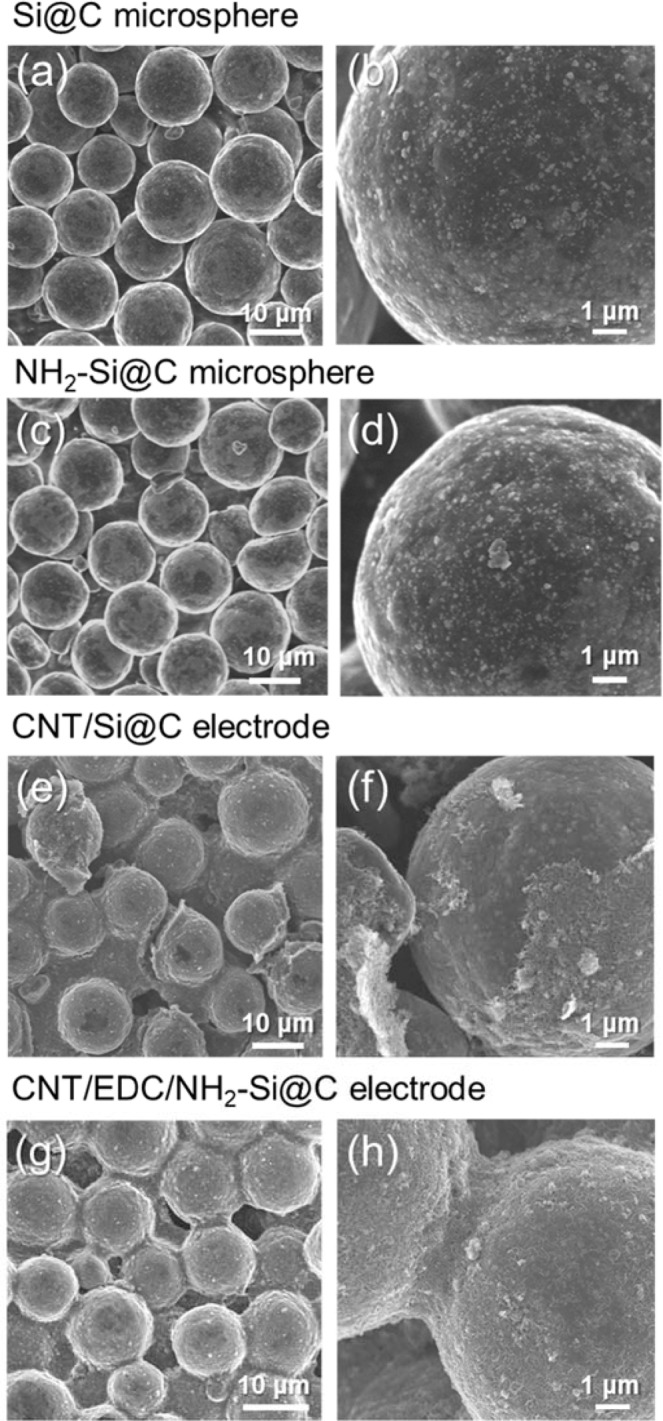
FE-SEM images of (**a**,**b**) bare Si@C particles, (**c**,**d**) NH_2_-Si@C particles, (**e**,**f**) non-covalently bound CNT/Si@C electrode (van der Waals interactions), and (**g**,**h**) covalently cross-linked CNT/EDC/NH_2_-Si@C electrode (covalent amide bond).

FE-SEM images show that the Si@C microspheres are highly uniform in shape. Detail structural characteristics were described in the literature^23^. For instance, the size distribution analyzer and nitrogen adsorption revealed their median size (D50) of 14.9 m and specific surface area of 1.4 m^2^/g, respectively. Their morphological characteristics remain same after the AHAMTES coating (Fig. 2a–d). This result implies that the AHAMTES layers were thinly coated on the surface. As shown in Fig. 2e–h, one-dimensional MW-CNTs homogeneously wrap the individual NH_2_-Si@C microsphere surface and are interwoven through the intergranular nanospace. The microspheres are intimately embedded in the three-dimensional (3D) electronic conductive network formed within the composite electrodes. These structural features are significantly different from those of conventionally used composite electrodes, while being very similar to those of binder-free supramolecularly assembled electrode systems, which we have demonstrated for NCM523 cathodes^21,22^. The thickness of MW-CNTs coating was averagely 200–300 nm, as evaluated by cross-sectional FE-SEM (Fig. S1). Physically attached MW-CNT seems to be containing, MW-CNTs in excess of those used for coating the microsphere surface were also consumed in the formation of junctions at the intergranular nanospaces. In contrast to the hybrid electrodes of MW-CNTs with NH_2_-EDC-Si@C microspheres (CNT/EDC/NH_2_-Si@C), the MW-CNT network in electrostatically interacting CNT/NH_2_-Si@C showed partial delamination from the NH_2_-Si@C microsphere surface. This difference is due to the lack of bond strength required to fix the MW-CNTs to the entire surface of the NH_2_-Si@C microsphere. The incorporation of amide bonds at the junction primarily contributes to the stabilization and reinforcement of MW-CNTs in the covalently bound CNT/EDC/NH_2_-Si@C hybrid electrodes.

### Electrochemical performance of the composite electrodes

**Figure 3:**
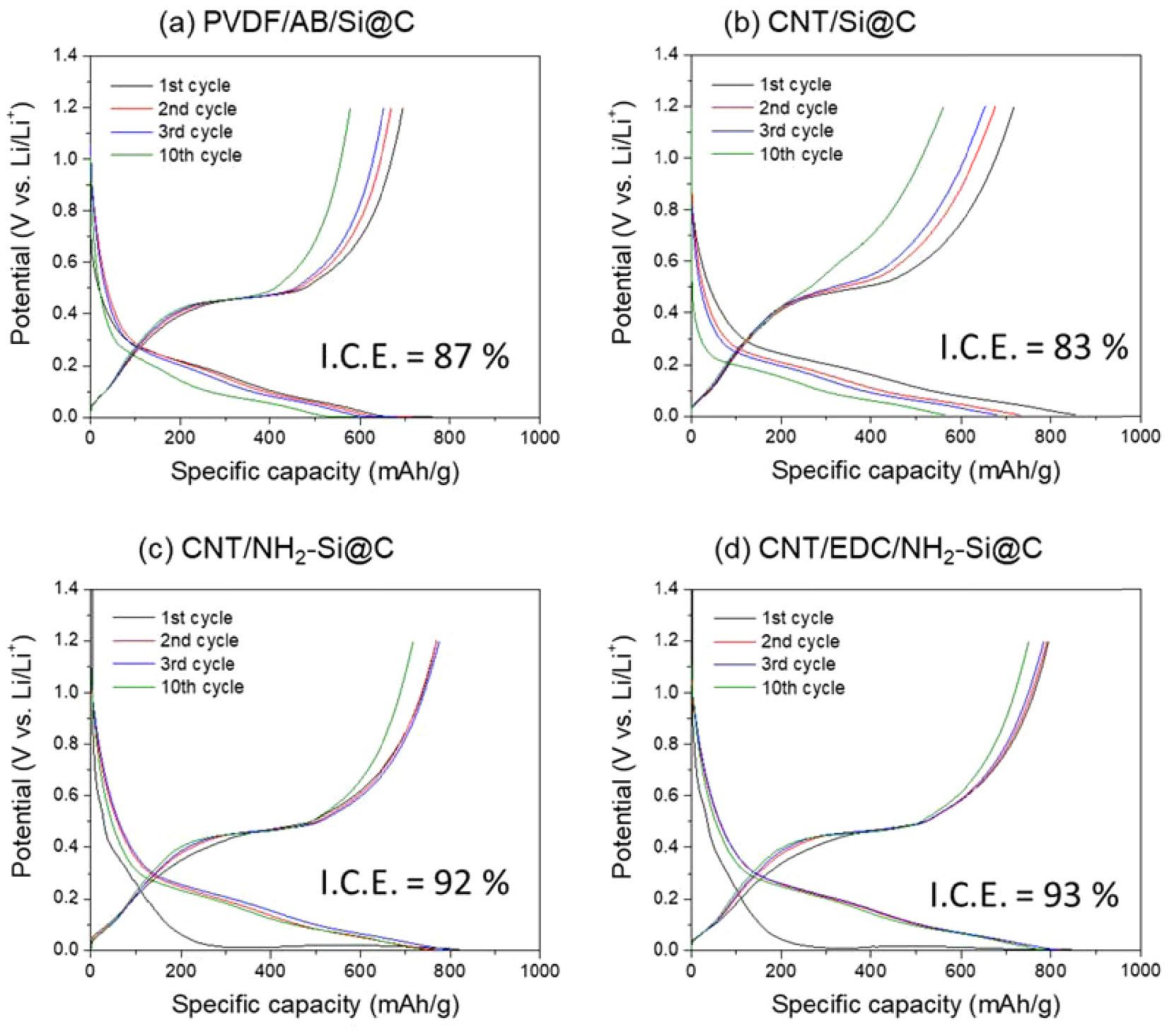
Galvanostatic charge-discharge profiles of Si@C-based electrodes at a current density of 0.5 A g^−1^ between 0.005 and 1.2 V (vs. Li/Li^+^): (**a**) PVDF/AB/Si@C, (**b**) CNT/Si@C (**c**) CNT/NH_2_-Si@C, and (**d**) CNT/EDC/NH_2_-Si@C.

To demonstrate the effect of EDC coupling on the electrochemical performance of the composite electrode, half-cells composed of CNT/Si@C, CNT/NH_2_-Si@C, and CNTs/EDC/NH_2_-Si@C were tested and the results are compared. Figure 3 shows the first ten consecutive galvanostatic charge-discharge profiles of all the electrodes tested in 2032 type coin cells. No polymeric binder was used in the preparation of electrodes to verify the effect of amide bonding on the robustness of the MW-CNT micro-grid network. Hybrid electrodes based on CNT/Si@C microsphere delivered an initial capacity of 830–855 mAh g^−1^ in total mass equivalent of the Si@C microsphere and ~2441–2514 mAh g^−1^ in total mass equivalent of the nano-Si crystal, respectively. Surface functionalization with AHAMTES decreased the initial capacity of the microsphere, which is reasonable because the capacity is calculated based on the total mass of the composite. The weight of the Si@C microsphere increased with the incorporation of AHAMTES and EDC, which resulted in decreased capacity of the material. A system based on acetylene black and PVDF binder (PVDF/AB/Si@C) was also tested for further comparison. The PVDF/AB/Si@C electrode was found to have the lowest initial capacity of 730 mAh g^−1^ in the first lithiation process, which suggests the lack of electron conductivity in the electrode due to weaker interaction between PVDF and Si@C microspheres.

**Figure 4:**
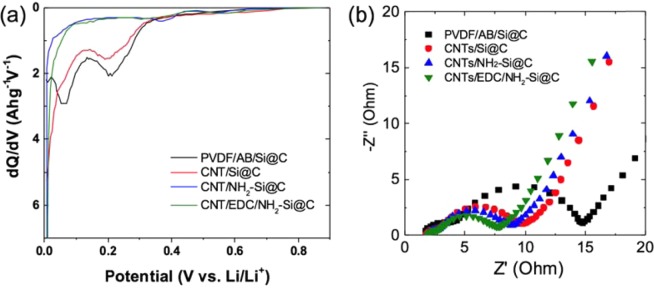
(**a**) dQ/dV profiles of the series of half-cells of Si@C-based anodes at a current density of 0.5 A g^−1^ (first lithiation), and EIS spectra (**b**) after 5 cycles.

Note that the immobilization of an AHAMTES monolayer on the Si@C microsphere had further noticeable effects on the Coulombic efficiency in the first cycle for a given condition. Figure 4a presents the dQ/dV plots for the first cycle. The shape of the dQ/dV plots is different for different electrodes with the Si@C microspheres. The plots of PVDF/AB/Si@C and CNT/Si@C electrodes contain intense peaks at 0.21 and 0.07 V, which are likely due to both the reduction of the electrolyte as well as the silanol groups formed on nano-Si crystal surface (native oxide layer), respectively^20^. These peaks are not found in the plots of AHAMTES-monolayer-immobilized electrodes, suggesting that the AHAMTES monolayer altered the initial interfacial reactions. Since the capacity of the half-cell in the first lithiation process is associated with the reductive decomposition of electrolyte at the surface leading to form SEI formation and Li^+^ storage inside the nano-Si crystals, it can be concluded that the AHAMTES monolayer is responsible for passivating the nano-Si crystal surface exposed from the carbon microsphere as well as for reducing the specific surface area in direct contact with the electrolyte. This monolayer contributes to the mitigation of the SEI layer growth at the electrode-electrolyte interface. Very similar effects were observed in the high voltage spinel cathode surface with fluoroalkylsilane monolayer coating^24,25^.

According to the galvanostatic charge-discharge curves shown in Fig. 3, we further notice that the voltage profiles of the CNT/EDC/NH_2_-Si@C hybrid electrodes showed significantly lower polarization and small capacity fading during the first ten cycles as compared with those of other electrodes, indicating that the electron-conducting MW-CNT network is stiffly organized inside the electrode and remains mechanically robust during the cycles. The highly organized network of MW-CNT is considered to respond rapidly to lithiation/delithiation reactions with less polarization. Moreover, as shown in Fig. 4b and Table 1, the chemical impedance spectra acquired from the 5th cycle reveal that CNT/EDC/NH_2_-Si@C hybrid electrodes present the lowest surface and charge transfer resistances among the electrodes studied. The variation in the electrochemical properties according to the interaction between the MW-CNT network and Si@C suggests that physical interaction without any chemical bonding leads to interparticle connections with insufficient electron conductivity, whereas the coverage of the entire Si@C microsphere surface seemed to be same in all cases, as evaluated by FE-SEM observation, regardless of the type of interaction.

**Table 1:** EIS parameters of the series of the half-cells of Si@C-based anodes obtained after 5 and 50 cycles.

	*R*_SEI_ (Ω)		*R*_ct_ (Ω)	
	After 5	After 50	After 5	After 50
PVDF/AB/Si@C	2.76	4.73	9.92	42.61
CNT/Si@C	0.83	6.47	6.49	19.26
CNT/NH_2_-Si@C	0.99	5.62	4.51	16.98
CNT/EDC/NH_2_-Si@C	0.74	6.57	4.70	12.38

**Figure 5:**
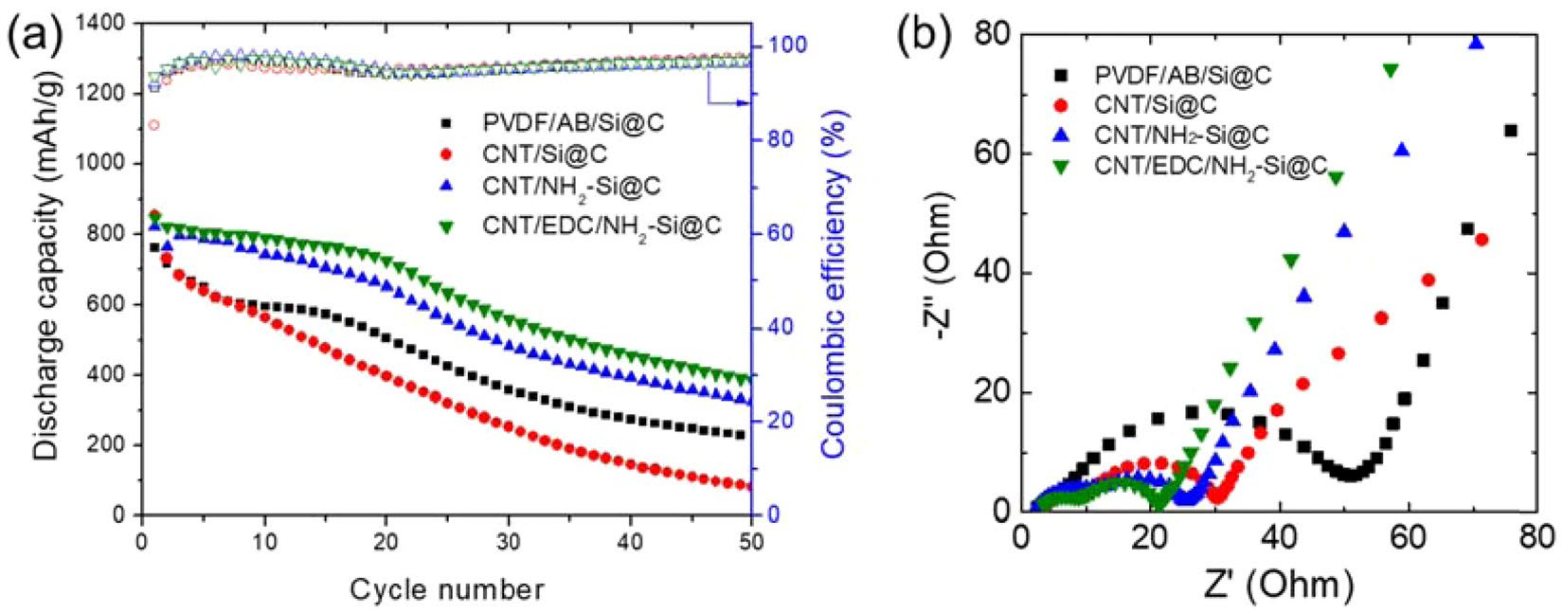
(**a**) Cycling performances of the half-cells of Si@C-based anodes at a current density of 0.5 A g^−1^ (50 cycles), and (**b**) the EIS spectra of cells after 50 cycles.

Cyclability tests were conducted to further investigate the effect of EDC coupling on the robustness of the CNTs/Si@C hybrid electrodes during cycling. Figure 5a shows the change in capacity as a function of cycle number, evaluated at a current density of 0.5 A g^−1^ for 50 cycles. PVDF/AB/Si@C and CNT-Si@C microsphere electrodes show significant decline in capacity during the initial stages of cycling. The reversible capacity degraded to 220 and 81 mAh·g^−1^ after 50 cycles, respectively. These values correspond to retention of ~28 and 10% of the initial capacity. It seems that the capacity drop was caused by the disconnection of the electron conductive network in the electrode accompanying the massive volume changes. CNT/EDC/NH_2_-Si@C microsphere electrodes showed remarkable improvements in both the specific capacity and Coulombic efficiency as compared with those of others, clearly demonstrating the benefit of introducing amide bonding for the fixation of MW-CNTs. The reinforcement of the MW-CNT network with covalent bonding led to an electron-conducting network that remained highly stable during large volumetric changes in nano-Si crystals corresponding to lithiation and delithiation reactions. A gradual capacity fading was still observed, a reversible capacity of ~400 mAh g^−1^ (capacity retention of ~47% with respect to that of the 1st cycle) was still maintained after 50 cycles, with an excellent average Coulombic efficiency of 99% throughout the cycles. The amide bond strengthened the bonding between Si@C and MW-CNT network, thereby improving the stability of the electron conduction network formed in the electrode. These results imply that van der Waals forces cannot provide sufficient binding force to firmly hold Si@C microsphere and CNT network together. Therefore, we attribute the maximum capacity retention observed for the CNT/EDC/NH_2_-Si@C microsphere electrode to stronger interaction between the two components of the composite, owing to amide linkage.

As shown in Fig. 5b, the Nyquist plots of cells subjected to 50 cycles at 25 °C consist of semicircles and a sloping line. The first semicircle in the high-to-medium frequency region corresponds to the resistance of the surface film (*R*_*Sf*_) that covers the Si@C microsphere, the second mid-frequency semicircle corresponds to the charge-transfer resistance (*R*_*ct*_) coupled with double layer capacitance, and the sloping line at the low-frequency region can be assigned to lithium-ion diffusion in the electrodes. The impedance spectra clearly display the contribution of the low-frequency element due to the modified SEI layer, which promotes Li^+^ transport through the immobilized AHAMTES monolayer. Furthermore, the impedance growth at low frequencies for CNT/EDC/NH_2_-Si@C is highly mitigated, as compared to those of CNT/NH_2_-Si@C and CNT/Si@C. This result indicates the contribution of the formation of a robust 3D electron conduction pathway inside the electrode, owing to stronger interaction between the MW-CNT network and NH_2_-Si@C, because of EDC coupling. The C rate capabilities of CNT/Si@C, CNT/NH_2_-Si@C, and CNT/EDC/NH_2_-Si@C electrodes were shown in Fig. S2. Irreversible capacity loss has been observed in the discharge reaction due to imperfect inhabitation to the volume change of electrodes during the cycles (details were described below), however the formation of amide bonds clearly contributes to the improvement of kinetical properties.

**Figure 6:**
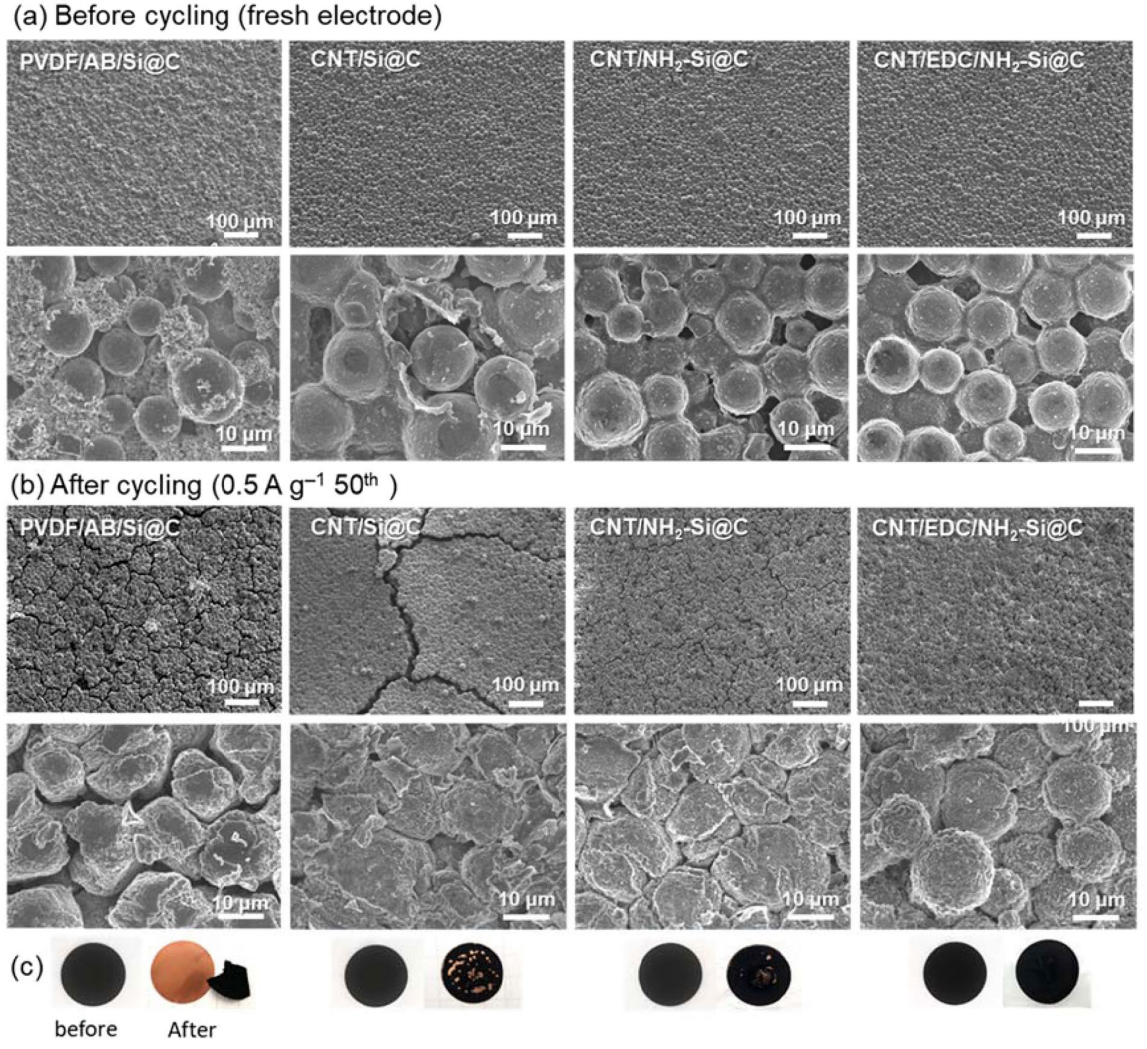
FE-SEM images showing the surface microstructures of PVDF/AB/Si@C, CNT/Si@C, CNT/NH_2_-Si@C, and CNT/EDC/NH_2_-Si@C electrodes (**a**) before (fresh electrode) and (**b**) after 50 cycles. (**c**) The corresponding digital images of cycled electrodes.

In order to confirm the structural stability driven by strong interaction between the components of the electrode, changes in surface morphologies during cycling were characterized by FE-SEM. Figure 6a shows the surface FE-SEM images of pristine electrodes. The Si@C microspheres and acetylene black are inhomogeneously distributed in the PVDF/AB/Si@C electrode. In contrast, CNT/Si@C hybrid systems seem to display different morphological characteristics. All the electrodes are well-structured and do not show any critical defects, even in the absence of polymeric binders. The MW-CNTs are homogeneously distributed over the Si@C microsphere surface, regardless of chemical bonding at the interface. Each microsphere is closely bridged by the MW-CNTs, leading to the formation of a micro-grid network in the electrodes. The morphological characteristics of the hybrid electrodes are very similar to those of the supramolecular assemblies of MW-CNT/NMC523 cathodes reported previously^21,22^. This suggests that short MW-CNTs are preferentially consumed by adsorption to the microsphere surface. Subsequently, the excess MW-CNTs are considered to self-aggregate at the intergranular nanospaces, assisted by the shear force acting on the NMP paste during the coating process. Note that the MW-CNT layer was partially peeled from the CNT/Si@C electrode surface. Since no defects are found in either CNT/NH_2_-Si@C or CNT/EDC/NH_2_-Si@C, it can be reasonably concluded that the introduction of chemical interaction at the interface significantly impacted the structural durability against the shear force during the coating process.

The robustness of the interface design is clearly reflected in the morphological degradation characteristic of the hybrid electrode after cycling (Fig. 6b). Some cracks and defective microspheres are observed in the all electrodes, depending on the interface design for forming the hybrids. The PVDF/AB/Si@C electrode showed a very different morphology with a large number of cracks and small debris, probably due to the weak interaction of PVDF with the Si@C microspheres. High-magnification images reveal complete deformation of individual microspheres after cycling. Furthermore, a thick SEI layer is observed on the microsphere surface, which is attributed to the continuous side reactions occurring at the interface, as well as the volume expansion of Si particles during the charge and discharge cycles. In contrast, CNT/Si@C electrodes containing the MW-CNT binder show larger cracks compared to those of the PVDF/AB/Si@C electrode, although the extent of the deterioration of individual Si@C microspheres in the electrodes is very similar to those of PVDF-based electrodes. This result indicates that the MW-CNT binder is more vulnerable to large volume changes in nano-Si crystals because it is strongly integrated with the Si@C microsphere than with PVDF through the formation of a homogeneous 3D network. Very similar trends were shown in Si electrodes containing poly(acrylic acid) (PAA) binder by Nguyen *et al*. and Bridel *et al*.^20,26^. They reported that, as compared to the electrode containing the CMC binder, the highly interacting PAA binder rendered the nano-Si crystal-based electrode more rigid, leading to larger cracks after cycling. In contrast, the crack size decreased significantly in CNT/NH_2_-Si@C and CNT/EDC/NH_2_-Si@C electrodes as compared to those of others. Furthermore, the original morphological characteristics of the hybrid electrodes were maintained in CNT/EDC/NH_2_-Si@C electrodes at the individual microsphere level. Note that, as shown in Fig. 6c, except for the CNT/EDC/NH_2_-Si@C electrode, the electrodes are observed to have peeled from the copper current collectors after cycling. Further, although the cross-sectional FE-SEM images of the cycled electrodes revealed that the electrode thickness doubled for PVDF/AB/Si@C and CNT/Si@C electrodes, the increase in thickness was significantly suppressed for the CNT/EDC/NH_2_-Si@C electrodes (see Fig. S3). There is a high possibility that the flexibility of newly developed 3D-structured MW-CNT network is sufficient for mitigating the breakage of the electron-conducting network in the electrode due to the massive volume changes in the Si@C microsphere during the reversible lithiation/delithiation reaction. This might be due to the inherent flexibility of the supramolecularly assembled MW-CNT binder. This flexibility is amplified by the increased binding strength of MW-CNT to the Si@C microsphere owing to amide bond formation.

**Figure 7:**
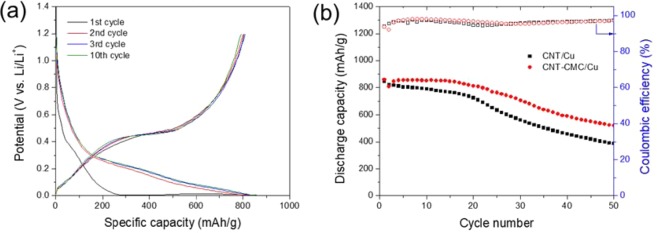
Effect of the modification of carbon-coated Cu current collector surface with CMC on the battery performance; (**a**) galvanostatic charge-discharge profiles of CNTs/EDC/NH_2_-Si@C using a modified current collector at a current density of 0.5 A g^−1^ and (**b**) the corresponding cycling performance.

A sudden capacity drop was observed for CNT/EDC/NH_2_-Si@C electrode after approximately 20 cycles, although there were no critical disconnections of electron conduction pathways along the MW-CNT grid. In order to enhance the binding strength between the electrode and Cu current collector at the interface, we modified the Cu surface with a carboxymethyl cellulose (CMC) binder. We coated a 9 wt% of mixed aqueous suspension of MW-CNT and CMC on the surface. The MW-CNT: CMC weight ratio was controlled to be 95:5 wt% in an aqueous solution. Galvanostatic charge-discharge test was conducted on the half-cell composed of CNT/EDC/NH_2_-Si@C electrode under the same conditions, except for the use of CMC-modified Cu current collector. Note that, as shown in Fig. 7, the capacity degradation curve sloped gently. The capacity after 50 cycles was obviously improved from 400 to 520 mAh.g^−1^. All these results indicate that the 3D MW-CNT network stretches to follow the large volume expansion and contraction of Si@C microspheres through the incorporation of amide bonds at the interface. In contrast, the adhesion strength of the MW-CNT to the Cu current collector was insufficient under the polymeric binder-free conditions. The lack of electron conductivity at the current collector interface was found to be one of the most powerful contributors to capacity degradation during cycling under present circumstances.

## Conclusion

We presented a strategic approach to improve the cycle performance of a polymeric, binder-free nano-Si crystal-based anode by incorporating a self-assembled monolayer and performing EDC coupling as a means of creating a robust molecular junction between the MW-CNTs and nano-Si@C microspheres. Hybrid electrodes based on the supramolecularly assembled MW-CNT network and Si@C microsphere will be one of the attractive candidates for enhancing the structural durability against volume changes in nano-Si crystals anode materials, as well as minimizing the impedance growth during the cycled battery operation of the electrode.

## Method

### Materials

Nano-silicon crystal-embedded soft-carbon microspheres (Si@C, TCP-207) and a 9 wt% aqueous suspension of multi-walled carbon nanotubes (TCW-261B, 12 nm diameter, 400–600 nm long), which were kindly supplied by TODA KOGYO CORP., were used as precursors for the preparation of MW-CNT/Si@C hybrid electrodes. Average diameter of the Si@C microsphere is ~12 μm. The soft carbon microspheres were prepared by a thermal chemical vaporization deposition (CVD) process^26^. Approximately 34 wt% of nano-silicon crystals (~50–200 nm in size) were homogeneously distributed in each microsphere (Fig. S4). MW-CNTs remained stably suspended in an aqueous suspension without the need for agitation even after two years since the preparation of the suspension. The length and width of the MW-CNTs were confirmed to be approximately 400–500 nm and 20 nm, respectively, through light scattering experiments (SALD-7500nano, SHIMAZU Co Ltd., Japan). The surface of the MW-CNTs was densely functionalized with hydrophilic groups, including carboxylic acid groups, during a cutting process in a 0.5 wt% aqueous carboxymethylcellulose (CMC) solution.

*N*-(6-Aminohexyl)aminomethyltriethoxysilane (C_13_H_32_N_2_O_3_Si, AHAMTES) and 1-ethyl-3-(3-dimethylaminopropyl)carbodiimide hydrochloride (C_8_H_18_ClN_3_, EDC-HCl) purchased from Tokyo Chemical Industry Co. Ltd., were used for the CNT assembly without further purification.

### Preparation of CNT/Si@C hybrid electrode

Immobilization of an amino-organosilane monolayer on the Si@C microsphere: Typically, 1 g of Si@C microsphere powder was added to 50 mL of ethanol containing 200 μL of AHAMTES. The mixture was reacted at room temperature overnight to obtain AHAMTES-functionalized Si@C (NH_2_-Si@C). Formation of covalently cross-linked CNT network and Si@C: To enhance the interaction between the carboxylic acid-functionalized MW-CNTs and NH_2_-Si@C microspheres, water-soluble EDC, known as a zero-length carbodiimide crosslinker^27^, was used for the activation of the carboxyl group to enable its spontaneous coupling with a primary amine. These chemical reactions were performed during the slurry preparation process. First, for activating the carboxyl groups, 0.44 g of a 9 wt% aqueous suspension of MW-CNT and 40 μL of a 50 mM aqueous solution of EDC were mixed well using a planetary centrifugal mixer (AR100, THINKY). Then, 90 wt% of the NH_2_-Si@C powder was added and the resulting mixture was mixed until a homogeneous slurry was obtained (Table S1). Note that other active materials, conducting agents, or polymeric binders were not added to the slurry. The obtained slurry was coated on a carbon-coated copper foil using a Baker-type applicator with controllable coating speed (Eager Corp., Japan). The electrodes were dried at 80 °C for 12 h in a vacuum oven to evaporate the remaining water.

### Material characterization

The structure of the material was characterized using an X-ray diffractometer (XRD, Miniflex II, Rigaku, Japan) with Cu *Kα* (*λ* = 1.5418 Å) radiation, which was operated at 30 kV and 20 mA. The microstructures of the all the materials and electrodes were examined by field-emission scanning electron microscopy (FE-SEM, JSM-7600F, JEOL, Japan) at an accelerating voltage of 5 kV. X-ray photoelectron spectroscopy (XPS; JPS-9010MX, JEOL, Japan)) with a 150 W monochromatic Al *K*_*α*_ X-ray source (1486.6 eV). The XPS spectra were recorded in the binding energy range of 0 to 1400 eV at a step size of 1 eV and pass energy of 160 eV. High-resolution spectra were obtained at a step size of 0.1 eV. Fourier-transform infrared spectroscopy (FT-IR, JASCO FT/IR-6100 series, Japan) with KBr pellet method was conducted to analyze the chemical bonding of the material. The all operations for taking the spectra were performed under vacuum.

### Electrochemical characterization

The electrodes were assembled into 2032 type coin cells with 1 mol L^−1^ LiPF_6_ in a mixture of ethylene carbonate-dimethyl carbonate (1:2 ratio by volume) (Kishida Chemical Co. Ltd.) as the electrolyte. The loading amount of Si@C microsphere was controlled at ~1.5 mg·cm^−2^. Lithium metal (99.9%, Honjyo Metal Co. Ltd.) was used as the counter electrode and Celgard 2400 membrane (Celgard Inc., USA) was used as a separator. In order to verify the effects of covalently cross-linked MW-CNT networks on the robustness of Si@C-based electrodes, the following electrodes were also prepared for comparison: (i) CNT/Si@C (obtained by mixing two components without any surface modification; physisorption), (ii) CNT/NH_2_-Si@C (prepared by mixing MW-CNTs and amino-organosilane-functionalized Si@C microspheres; electrostatic attraction), and (iii) Si@C/AB/PVDF (a conventional electrode system prepared using Si@C, acetylene black, and polyvinylidene fluoride (PVDF) binder at a mass ratio of 80:10:10 in *N*-methyl-2-pyrrolidone (NMP) solvent). Galvanostatic charge–discharge tests were performed using a potentiostat/galvanostat (HJ1020Msd8, Hokuto Denko, Japan) in the potential window of 5 mV–1.2 V (vs. Li/Li^+^) at 25 ± 1 °C in a constant-temperature chamber (SU-221, Espec). The current density was 0.5 A g^−1^. The capacities were calculated with respect to the total mass of the active material, including the coating layer. Electrochemical impedance spectroscopy (EIS) and cyclic voltammetry (CV) were carried out using a multi-potentiostat (VSP-300, Bio-Logic). The frequency range for the EIS measurements is 200 kHz to 1 mHz, and CV curves were obtained in the potential range of 5 mV–2.0 V (vs. Li/Li^+^) at a scan rate of 0.1 mV s^−1^.

## Supplementary information

Supplementary material.
