# Supplementary material for: Three-dimensional assembly of multiwalled carbon nanotubes for creating a robust electron-conducting network in silicon-carbon microsphere-based electrodes
Source: Sci Rep. 2020 Feb 11;10:2342. doi: 10.1038/s41598-020-58338-3 (PMC7012817; doi:10.1038/s41598-020-58338-3)
Supplement: Supplementary file 1 — Supplementary material. [file 41598_2020_58338_MOESM1_ESM.docx]

**Supporting information**

**Three-dimensional assembly of multiwalled carbon nanotubes for creating a robust electron-conducting network in silicon-carbon microsphere-based electrodes**

Hyemin Kim ^a^, Dae-wook Kim ^a^, Hitomi Todoki ^a^, Nobuyuki Zettsu *^a,b^, and Katsuya Teshima *^a,b^

^a^Department of Materials Chemistry, Faculty of Engineering, Shinshu University, 4-17-1 Wakasato, Nagano 380-8553, Japan.

^b^Research Initiative for Supra-Materials, Shinshu University, 4-17-1 Wakasato, Nagano 380-8553, Japan.

E-mail: zettsu@shinshu-u.ac.jp and teshima@shinshu-u.ac.jp

**Table S1**. Experimental conditions for the preparation of various electrodes

| Type | Interaction | Active materials | Conductive agents | Binder |
| --- | --- | --- | --- | --- |
|  |  | Si@C (wt%) | MW-CNTs in H_2_O (wt%) | (wt %) |
| (a) CNT/Si@C | van der Waals force | 90 | 10 | – |
| (b) CNT/NH_2_-Si@C | Electrostatic attraction | 90 | 10 | – |
| (c) CNT/EDC/NH_2_-Si@C | Covalent bonding | 90 | 10 | – |
| (d) PVDF/AB/Si@C | – | 80 | 10 (AB) | 10 (NMP) |





**Figure S1**. Cross-sectional FE-SEM images of CNT/EDC/NH_2_-Si@C electrode.


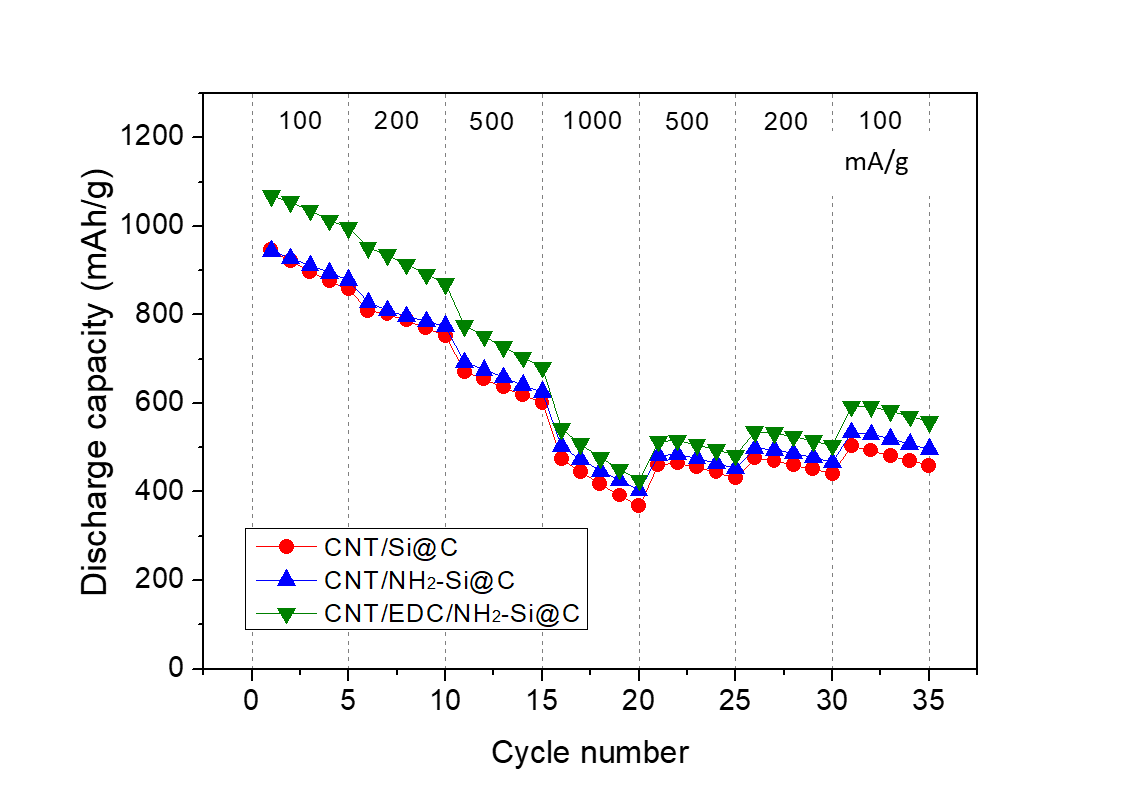


**Figure S2**. Discharge capacities of the half-cells of Si@C-based electrodes at a various current density ranging from 0.1 A·g-1 to 1.0 A·g-1.


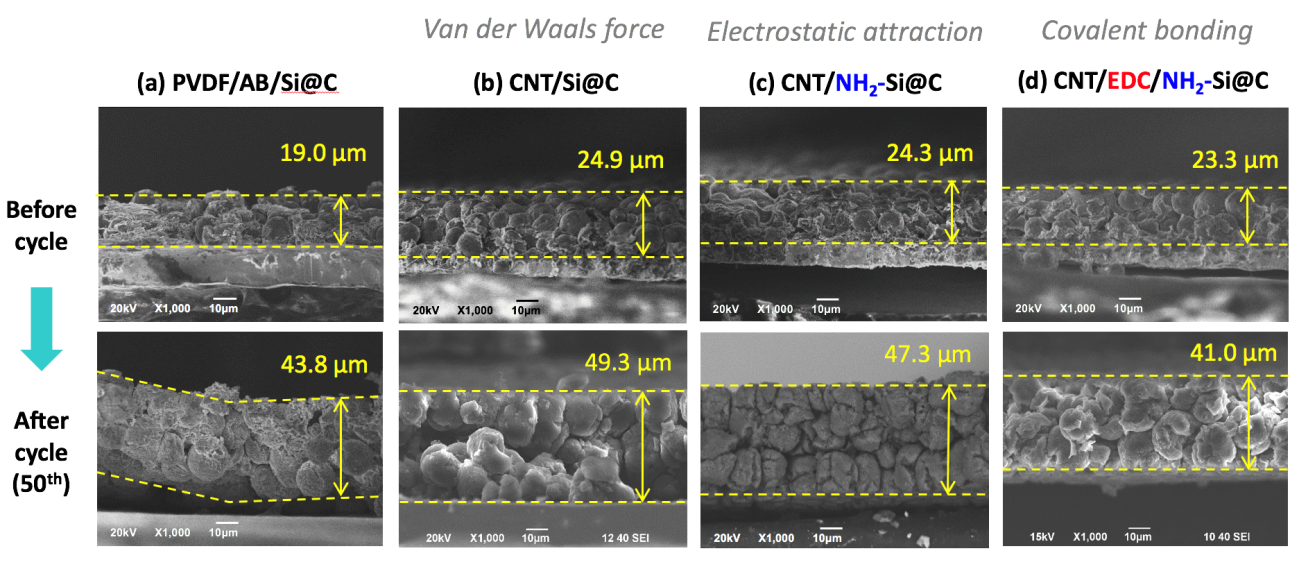


**Figure S3**. Cross-sectional FE-SEM images of cycled Si@C-based anodes: (a) PVDF/AB/Si@C, (b) CNT/Si@C, (c) CNT/NH_2_-Si@C, (d) CNT/EDC/NH_2_-Si@C.

**
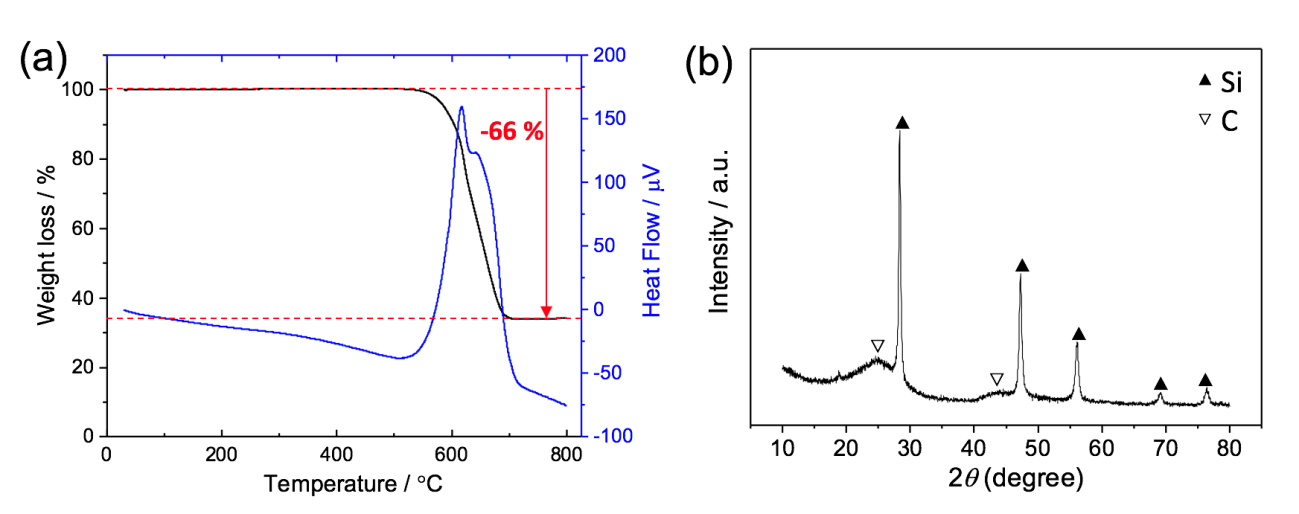
**

**Figure S4**. (a) TG-DTA profiles and (b) XRD patterns of as received Si@C particles.
